# Melanoma Arising in a Medium-Sized Congenital Melanocytic Nevus: A Case Report

**DOI:** 10.7759/cureus.109026

**Published:** 2026-05-17

**Authors:** Kazutoshi Nishimura, Yuto Yamamura, Shunya Usui, Kazuyasu Fujii, Atsushi Otsuka

**Affiliations:** 1 Department of Dermatology, Kindai University Hospital, Osaka, JPN

**Keywords:** congenital melanocytic nevus, malignant melanoma, malignant transformation, medium-sized nevus, prame

## Abstract

Congenital melanocytic nevus (CMN) is a melanocytic lesion present at birth or in early infancy, and most cases follow a benign clinical course. While the risk of malignant melanoma (MM) is known to be increased in giant CMN, the risk in small- to medium-sized CMN is considered low, and routine prophylactic excision is generally not recommended.

We report a case of MM arising in a medium-sized CMN in a 23-year-old woman. The lesion had remained stable for many years but showed rapid enlargement and bleeding over a two-month period. Histopathological examination revealed nodular proliferation of atypical melanocytic cells with marked nuclear pleomorphism, high mitotic activity, and vascular invasion. Immunohistochemical analysis demonstrated diffuse Preferentially expressed Antigen in MElanoma positivity, a high Ki-67 labeling index, and loss of p16 expression. Based on the integrated evaluation of morphological and immunohistochemical findings, a diagnosis of MM arising in CMN was made. The tumor was classified as Stage IIc (T4bN0M0) according to the American Joint Committee on Cancer Eighth edition.

This case highlights that even in small- to medium-sized CMN, rapid clinical changes such as enlargement and bleeding may represent important warning signs of malignant transformation. Careful clinical monitoring and appropriate patient education are essential for the early detection of MM in such lesions.

## Introduction

Congenital melanocytic nevus (CMN) is a melanocytic lesion present at birth or in early infancy [1]. CMN is classified according to its projected adult size, based on established dermatological consensus criteria proposed by Krengel et al. [2], into small (<1.5 cm), medium (1.5-20 cm), and giant (>20 cm). The majority of cases follow a benign clinical course throughout life.

Malignant melanoma (MM) is an aggressive skin cancer arising from melanocytes, characterized by a high metastatic potential and a prognosis that largely depends on the stage at diagnosis. The five-year survival rate exceeds 90% in early-stage disease but decreases markedly in advanced stages. Although MM most commonly occurs in older individuals, it is relatively rare in young adults, and cases associated with preexisting melanocytic lesions such as congenital melanocytic nevi are uncommon [3].

In contrast, giant congenital melanocytic nevus (GCMN) is associated with an increased risk of MM, and in some cases, proactive management, including prophylactic excision, may be considered [4].

In small- to medium-sized CMN, however, the risk of MM is considered to be low, and careful clinical observation with attention to changes over time is generally recommended as the standard management approach [4]. Clinically, changes such as rapid enlargement, nodular development, bleeding, or ulceration are considered important warning signs that warrant further evaluation. In routine clinical practice, many small- and medium-sized CMN remain untreated for long periods and are often not regarded as problematic by patients.

Herein, we report a case of MM arising in a medium-sized CMN located on the anterior chest of a young woman, which was diagnosed following rapid enlargement and bleeding over a short period. Reports of MM arising in medium-sized CMN are limited. This case highlights the importance of dermatological follow-up of CMN and emphasizes that rapid clinical changes may serve as important warning signs of malignant transformation.

## Case presentation

A 23-year-old woman has had a CMN with hair growth on her upper abdomen since birth. Although she had no history of trauma, she was referred to our department because the lesion had rapidly expanded over the past two months. She had no significant past medical or surgical history, had no relevant family history, and was not taking any regular medications.

**Figure 1:**
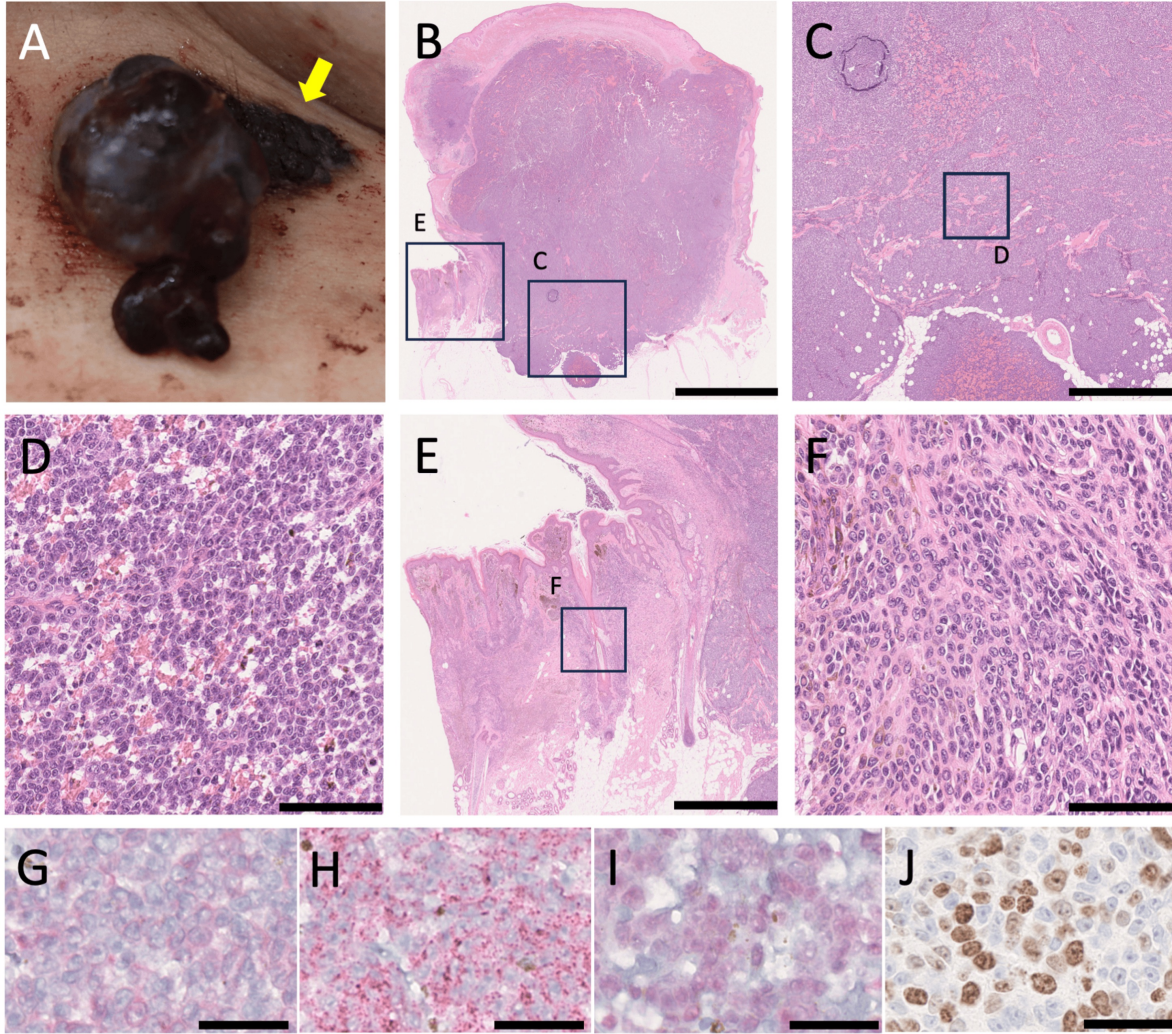
Clinical and histopathological findings (A) A 40 × 28 mm hairy brown plaque on the epigastric region with a 25 × 25 mm pedunculated, easily bleeding black nodule within the lesion. The arrow indicates the background congenital melanocytic nevus. (B) Low-power view showing nodular proliferation of tumor cells extending from the epidermis with ulceration into the subcutaneous tissue. The boxed area corresponds to the higher magnification views in (C-E). (C,D) Tumor cells contain a small amount of melanin and show marked nuclear pleomorphism with prominent nucleoli, frequent mitotic figures, and vascular invasion. (E,F) The peripheral area of the nodular lesion shows maturation of nevus cells and extension around adnexal structures. (G) Diffuse positivity for Melan-A. (H) Diffuse positivity for HMB45. (I) Diffuse positivity for PRAME. (J) Ki-67 labeling index of approximately 50%. Scale bars: 250 µm (B,C,E); 100 µm (D,F); 50 µm (G-J) PRAME: Preferentially expressed Antigen in MElanoma

At the initial visit, a 40 × 28 mm hairy brown plaque was noted in the upper abdomen, containing a 25 × 25 mm pedunculated black nodule that bled easily (Figure 1A). Due to recurrent bleeding, an excisional biopsy was performed. Following a histopathological diagnosis of MM, a wide local excision with a 10-mm margin was performed one week later. Histopathological examination confirmed negative surgical margins.

Histopathological examination revealed nodular proliferation of tumor cells from the epidermis into the subcutaneous tissue, accompanied by ulceration (Figure 1B). The tumor cells contained a small amount of melanin and exhibited marked nuclear pleomorphism, distinct nucleoli, frequent mitotic figures, and vascular invasion (Figures 1C, 1D). A compound nevus component was observed at the margins of the nodules (Figures 1E, 1F).

Immunohistochemically, the tumor cells were positive for Human Melanoma Black-45 and Melan A, and showed diffuse positivity for Preferentially expressed Antigen in MElanoma (PRAME). The Ki-67 index was approximately 50%, and loss of p16 expression was observed (Figures 1G-1J). Based on a comprehensive evaluation of the morphological and immunohistochemical findings, the diagnosis was MM arising from a CMN. The Breslow thickness was 19 mm.

Preoperative lymphoscintigraphy identified bilateral axillary sentinel lymph nodes, and sentinel lymph node biopsies were performed in both axillary regions during wide local excision. No metastasis was detected in any of the lymph nodes, and no evidence of distant metastasis was found on whole-body CT. Based on the Eighth edition of the American Joint Committee on Cancer, the tumor was classified as Stage IIc (T4bN0M0).

Adjuvant therapy is recommended for stage IIc melanoma with negative surgical margins. For patients with melanoma harboring the BRAF V600E mutation, BRAF/MEK inhibitors or anti-PD-1 therapy are options for adjuvant therapy. In this case, the BRAF V600E mutation was confirmed, and the patient is currently receiving adjuvant therapy with a BRAF/MEK inhibitor.

## Discussion

The association between CMN and MM has been well documented, particularly in patients with severe congenital phenotypes, including GCMN and multiple CMN, in whom an increased risk of MM has been reported [5]. In contrast, the overall risk of MM arising in small- to medium-sized CMN is considered low, and these lesions are not regarded as premalignant [4]. Accordingly, prophylactic excision is generally not recommended for small- and medium-sized CMN, and management is typically based on careful clinical observation with attention to changes over time [4]. However, risk assessment based solely on lesion size may not be sufficient to identify all cases at risk of malignant transformation, and dynamic clinical changes such as rapid enlargement, nodular development, bleeding, or ulceration should also be considered as important indicators warranting further evaluation.

The present case is characterized by the development of MM in a medium-sized CMN (approximately 3-4 cm) in a young adult. Most reported cases of MM associated with CMN have been observed in patients with GCMN or in pediatric populations, and adult cases arising in medium-sized CMN are rare in the literature [6]. In addition, the lesion in this case had remained clinically stable for many years but exhibited rapid nodular growth and bleeding within a short period of two months, which represents an important clinical feature.

Clinically, lesion size is an important risk factor in the management of CMN; however, temporal morphological changes have also been reported as important warning signs [7]. Clinical features such as rapid enlargement, nodular formation, bleeding, and ulceration are highly significant for distinguishing benign from malignant lesions. Even in small- or medium-sized CMN, the presence of these changes should lead to prompt evaluation and consideration of excision [7]. The present case further suggests that careful attention to clinical changes is warranted even in long-standing, clinically stable, flat CMN.

On the other hand, some cases of MM arising in CMN may be difficult to distinguish from proliferative nodules (PNs) or borderline lesions [7]. PNs developing within CMN can exhibit increased cellular density, nuclear atypia, and mitotic activity, and may closely mimic MM both clinically and histopathologically. Previous studies have also highlighted the diagnostic challenges, even with long-term follow-up [8]. In the present case, marked nuclear atypia and nodular growth with an irregular proliferation pattern were observed on histopathological examination. In addition, diffuse PRAME positivity was demonstrated on immunohistochemical analysis. Based on the integrated evaluation of these morphological and immunohistochemical findings, a diagnosis of MM rather than PN was established [9].

## Conclusions

This case represents MM arising in a medium-sized CMN. Although routine prophylactic excision is not recommended for small- to medium-sized CMN, such lesions should be included in dermatological follow-up, and patients should be adequately informed about the importance of clinical changes. Even in lesions considered to be low risk, early detection of changes through regular observation and patient self-monitoring may contribute to the early diagnosis of MM.
